# Stress and Coping Strategies of Hong Kong University Students During the COVID-19 Pandemic: A Qualitative Study

**DOI:** 10.3390/ijerph22091359

**Published:** 2025-08-29

**Authors:** Tingyin Wong, Daniel T. L. Shek

**Affiliations:** Department of Applied Social Sciences, The Hong Kong Polytechnic University, Hong Kong 999077, China; tingyinwong@gmail.com

**Keywords:** coping, university students, COVID-19 pandemic, qualitative, thematic analysis

## Abstract

The COVID-19 pandemic brought significant challenges to university students in China, including the Hong Kong Special Administrative Region. To understand the stress and coping strategies of university students during the pandemic, we conducted focus groups with 56 Hong Kong university students from late December 2022 to mid-January 2023. Thematic analysis using a deductive data analytic approach based on the Transactional Theory of Stress and Coping was applied to form concepts on coping strategies. The findings revealed four major challenges faced by Hong Kong university students, which were the accumulation of negative emotions, health-related anxiety and frequent change in pandemic-related policies, conflict with family members, and challenges in online learning and academic and career development. When coping with these challenges, students used the following coping strategies: (1) seeking social support, emphasizing the positive and tension reduction to manage their negative emotions; (2) problem-focused coping and emphasizing the positive to deal with health-related anxiety and stress arising from the frequent change in pandemic-related policies; (3) seeking social support, tension reduction, distancing/detachment and self-isolation/keeping to themselves to handle conflict with family members; (4) seeking social support, problem-focused coping, emphasizing the positive and using distancing/detachment to cope with challenges in online learning and academic and career development. Moreover, comparing students with different backgrounds, the findings showed that more students with a high level of self-perceived resilience employed the emphasizing the positive coping strategy, while more students with financial difficulties applied tension-reduction coping strategies. This study contributes to the stress and coping literature by illustrating Hong Kong young people’s stress and coping strategies during the COVID-19 pandemic. This study also supports the Transactional Theory of Stress and Coping and extends the discussion to various coping theories.

## 1. Introduction

The COVID-19 pandemic has significantly influenced individuals’ lives and well-being. In Hong Kong, the first COVID-19 infection case was reported in January 2020. Since then, the city has experienced five waves of the pandemic. The number of daily reported infection cases was highest in March 2022. By the end of January 2023, there had been more than 1.22 million positive nucleic acid test reported cases and 1.88 million positive rapid antigen test reported cases [1]. Hong Kong university students experienced various challenges during the pandemic. Many activities related to their university studies needed to be postponed, including graduation ceremonies and study exchanges, which led to the loss of social life and deteriorated mental well-being. Moreover, online learning posed significant challenges for university students. Studies showed that online learning caused emotional distress in students through the lack of feedback and clarity in course arrangements [2], difficulties in teacher–student and student–student interactions [3], and technical problems during online assessment [4].

Studies conducted during the pandemic highlighted the substantial influence of various pandemic-related challenges on Hong Kong university students’ mental well-being. Shek et al. [5] revealed that there was a high percentage of university students with moderate or above levels of depression, anxiety and stress (depression: 40.0%, anxiety: 50.7%, stress: 22.2%). Lai et al. [6] also found that 39.4% Hong Kong university students had anxiety symptoms, while 32.6% had depression symptoms, during the pandemic. However, a review of the literature shows that most studies on young people are epidemiological studies on mental health problems, and research on their coping strategies is limited. In Hong Kong, a few researchers investigated the use of coping strategies by university students during the pandemic. Chan et al. [7] found that 81.2% of healthcare students used approach-based coping strategies, but more than a quarter of nursing students employed avoidance-based coping strategies during the pandemic. The application of approach-based coping strategies was associated with better psychological well-being. Analyzing university students in Hong Kong and Mainland China collectively, Cheng et al. [8] found that wishful thinking and empathetic responses predicted students’ compliance with the social distancing and personal hygiene measures. The mixed-methods study by Lai et al. [6] showed that Hong Kong university students’ most favored sources and means of support were peer support and phone contacts, while updated university guidelines were the most useful form of university support.

Moreover, while some studies investigated how university students coped with challenges related to pandemic-related precautionary measures, travel restrictions, fear of infection or online learning challenges, relatively few studies examined how they coped with financial difficulties. Patias et al. [9] studied Brazilian undergraduate students during the pandemic and found that low family income was associated with more depression, anxiety and stress symptoms and the use of positive re-evaluation strategies. Focusing on low-income university students in New York, Rudenstine et al. [10] found that both trauma-focused coping (finding meaning in trauma or stressors) and forward-focused coping (planning for the future) moderated the association of COVID-19-related stress with anxiety. In Hong Kong, the loss of part-time jobs because of the pandemic may have affected university students’ financial stability [11]. Studies showed that individual and family financial difficulties, unemployment and living alone correlated significantly with mental distress in Hong Kong university students [12], while positive family functioning and Chinese cultural beliefs of adversity were protective factors for students’ mental health problems [13].

Moreover, studies on the challenges and coping strategies of international college students during the pandemic showed that international students in different contexts experienced distinct challenges and applied diverse coping strategies. Examining international university students studying in the UK and US, Lai et al. [14] found that international students who stayed in the country where they attended university experienced more mental distress than students who returned to their home country. Resilience, positive thinking and physical exercise significantly predicted better mental health in international college students. Investigating Chinese college students studying in different foreign countries, Xia and Duan [15] found that active coping and self-adjustment were preferred by these students to cope with stress during the pandemic. Identification with Chinese culture beliefs also helped these students to apply positive strategies to cope with such challenges. In Hong Kong, Mainland Chinese students constitute a significant percentage of overall undergraduate students. Interestingly, Shek et al. [16] found that local students in Hong Kong suffered from more depressive symptoms than international students because of the cumulative stresses from social unrest and COVID-19 and the small living space in Hong Kong, which created challenges for students during home confinement. Wang et al. [17] highlighted that language barriers and potentially conflicting political beliefs created additional challenges for local and international university students in Hong Kong to build good relationships with each other. Findings from the above literature showed that various ecological factors may have affected the challenges and coping strategies of university students during the pandemic. Hence, the current qualitative study could enrich our understanding of the situation among Hong Kong university students.

Promoting resilience is one of the positive youth development constructs proposed by Catalano et al. [18], and resilience is defined as “an individual’s capacity for adapting to change and to stressful events in healthy and flexible ways” [18] (p. 102). Thus, resilient individuals are people who cope well during difficult times. Studies have revealed that university students with more resilience or positive attitudes are more likely to employ the coping strategy of reinterpretation [19] and adaptive coping strategies that include positive thinking [20]. This study further investigated how Hong Kong university students’ resilience was related to their coping attitudes and behaviors during the pandemic. Resilient university students’ coping was also compared with Mainland Chinese students and students with financial difficulties.

Although numerous studies were conducted during the pandemic, most research focused on the mental problems of young people, while their coping strategies were not sufficiently investigated. Moreover, there were insufficient qualitative studies on the topic. A literature map consolidating the literature presented and identifying the literature gap is shown in Figure S1. This study could fill the knowledge gap by qualitatively exploring the challenges and coping strategies of Hong Kong university students. Furthermore, we compared the coping strategies of resilient students, students with financial difficulties and Mainland Chinese students studying in Hong Kong, who are not given much attention in the literature. Based on the deductive analytic approach [21,22], this study applied the Transactional Theory of Stress and Coping to explore specific coping attitudes and behaviors. The following research questions were explored:

1. How did Hong Kong university students cope with stress and challenges during the COVID-19 pandemic?
2. What were the differences in coping strategies among students with high levels of self-perceived resilience, students with financial difficulties and Mainland Chinese students studying in Hong Kong?

## 2. Theoretical Framework

Coping is defined as the “constantly changing cognitive and behavioral efforts to manage specific external and/or internal demands that are appraised as taxing or exceeding the resources of the person” [23] (p. 141). The Transactional Theory of Stress and Coping describes four key components in the coping process: (1) primary appraisal, (2) secondary appraisal, (3) coping and (4) adaptational outcomes. The 68-item Ways of Coping Checklist (WCCL) was developed through an in-depth literature review and self-derived theoretical framework by the researchers in [24]. Items in the WCCL were originally divided into either problem-focused or emotion-focused coping. Problem-focused coping refers to the cognitive efforts used in problem-solving and behavioral strategies for handling the cause of problems, while emotion-focused coping refers to the cognitive and behavioral efforts for reducing negative emotions [25]. A subsequent study conducted by Folkman and Lazarus [26] studied undergraduate students’ coping during exams and revised the original WCCL into eight scales using factor analysis, including one problem-focused scale, six emotion-focused scales (i.e., wishful thinking, emphasizing the positive, tension reduction, self-blame, distancing, and self-isolation) and one mixed problem/emotion-focused scale (i.e., seeking social support). Items in these scales describe the cognitive and behavioral strategies that individuals apply to manage stressful situations. This study used these items to identify research participants’ coping strategies. This analytic approach aligns with the arguments made by researchers on stress and coping. For example, Skinner et al. [27] proposed that coping instances constitute ways of coping, families of coping strategies and adaptive processes, while Compas et al. [28] suggested that there are three levels of coping (domains, factors and strategies). This study focused on the “strategies” level by analyzing research participants’ thoughts and actions related to coping with stress during the pandemic. The eight Ways of Coping Scales guided the analytical process.

In addition to problem-focused coping and emotion-focused coping, other coping dimensions were proposed by researchers. One of the most widely used instruments is the Brief COPE Inventory [29], which was developed based on the WCCL and the model of behavioral self-regulation [30], dividing coping strategies into adaptive and maladaptive ones [31]. Moreover, the Kidcope scale [32,33] divides coping strategies into avoidant (e.g., social withdrawal), negative (e.g., self-criticism) and active (e.g., problem solving) coping strategies, while the Simplified Coping Style Questionnaire (SCSQ) [34] categorizes coping into active (i.e., responding actively to setbacks) and passive (i.e., responding passively to setbacks) coping strategies. Additionally, the Multidimensional Coping Inventory (MCI) [35] highlights three coping styles: task-oriented (e.g., adjust my priorities), emotion-oriented (e.g., daydream about a better time or place) and avoidance-oriented (e.g., see a movie) coping, while the Brief Approach/Avoidance Coping Questionnaire (BACQ) [36,37] analyses cognitive and emotional activities that are directed toward or away from threats. Furthermore, the Responses to Stress Questionnaire (RSQ) [38] divided coping mechanisms into primary control coping (i.e., changing environment based on self-desire) and secondary control coping (i.e., changing self to adapt to environmental factors). The above examples show that coping strategies can be grouped into subtypes of different coping dimensions based on factor analysis or conceptual analysis [39]. However, there are continuous debates and confusion over the underlying structure and subtypes of coping responses [40]. This study could fill in this knowledge gap by analyzing the research findings based on the Transactional Theory of Stress and Coping and extending the discussion to various coping theories.

The Transactional Model of Stress and Coping is regarded as a classical theory on coping. Many existing studies were stimulated by the WCCL [41]. Distinguishing coping into emotion-focused and problem-focused coping is an approach commonly adopted by researchers on coping [42,43]. During the COVID-19 pandemic, studies applied the Transactional Theory of Stress and Coping to study individuals’ coping strategies. Elkayal et al. [44] applied the WCCL to examine coping strategies used by the general population in Egypt during the pandemic. The study found that people with more severe mental distress adopted more ways of coping, and most people applied emotion-focused coping strategies to adapt to the situation during the pandemic. Li and Peng [45] also utilized problem-focused coping (including cognitive and behavioral coping) and emotion-focused coping concepts to study Chinese college students’ coping strategies during the pandemic, and they found high prevalence of cognitive coping, behavioral coping and social support but low prevalence of emotional-focused coping and anxiety in students. These studies demonstrated the applicability of the Transactional Theory of Stress and Coping to examining coping related to the COVID-19 pandemic.

Coping categories are the building blocks of coping research because they reflect the scope and substance of the field. However, one of the significant problems of constructing coping categories via factor analyses is the lack of clarity in category definitions [27]. Indeed, the eight scales of coping strategies were not defined by Folkman and Lazarus [26]. This study defined these eight scales by referencing the items in each scale and the definitions stated in another study conducted by Folkman et al. [46]. First, “distancing/detachment” denotes efforts made to detach oneself from the stressful situation. Second, “emphasizing the positive” refers to efforts made to create a positive outlook or positive meaning by focusing on personal growth. Third, “self-isolation/keep to self” describes efforts made to self-regulate feelings and actions. Fourth, “seeking social support” refers to efforts made to seek informational, tangible, emotional and religious support. Fifth, “wishful thinking” describes cognitive efforts based on fantasies or wishes to escape or avoid the stressful situation. Sixth, “problem-focused coping” refers to the deliberate efforts made to alter the stressful situation and the analytic approach made to solve the problem. Seventh, “tension reduction” describes the behavioral efforts based on tension reduction to reduce the stressful feeling. Eighth, “self-blame” refers to blaming oneself for the problem and trying to put things right.

This study employed a deductive analytic approach to investigate university students’ coping strategies during the pandemic. The eight Ways of Coping scales, which were developed based on the Transactional Theory of Stress and Coping, were used to formulate codes and themes regarding coping strategies.

## 3. Materials and Methods

### 3.1. Research Design

This study applied thematic analysis to the qualitative data analysis [47]. This study was underpinned by ontological relativism and epistemological post-positivism. This philosophical orientation stresses that there are multiple ways to understand reality and that knowledge is constructed based on continuous testing and revision [48,49]. The analytic process in thematic analysis could balance descriptive analysis, which summarizes trends or patterns in data, and interpretive analysis, which provides subjective analysis [22]. In this study, the post-positivist paradigm places more emphasis on the descriptions of objective reality. Moreover, researchers support the reliability of thematic analysis in deductive research because of the structured nature and theory-testing applications of both the post-positivist paradigm and the deductive analytic approach [50]. Researchers’ subjectivity is also highlighted as a resource in thematic analysis [47]. In this study, the authors’ reflexivity statements are presented in Appendix A.1.

Moreover, a deductive analytic approach was used in this study. There are several justifications for using a deductive approach in qualitative data analysis. First, instead of identifying themes emerging from the data, deductive qualitative analysis provides a more direct way to operationalize theories [21]. Second, a well-articulated theoretical framework can help to generate a more systematic picture of the findings. Although coping categories are the building blocks of coping research, there is a lack of consensus on the core coping categories in the literature, creating challenges for comparing and accumulating results and knowledge for further research [27]. Utilizing an existing theory in this study could resolve this issue by analyzing empirical data systematically [51]. Third, a deductive analytic approach can help to bridge the findings with existing theories and empirical findings. According to Boyatzis [52], one of the reasons for using theory-driven thematic coding is to replicate, extend or refute previous theories or findings. Thus, the application of theory-driven thematic analysis in this study could extend or refute the Transactional Theory of Stress and Coping.

### 3.2. Sampling and Procedures

From 29 December 2022 to 13 January 2023, students from one large-scale research university in Hong Kong were sampled for focus groups, which was during the period when Hong Kong was beginning to return to normal. A few local public universities had already returned to face-to-face teaching and learning starting from September 2022. From 29 December 2022, nucleic acid testing and social distance measures were canceled for people entering Hong Kong. Therefore, when students participated in this study, they were at the final stage of the pandemic and were starting to return to normal life. Compared with other countries or regions, Hong Kong kept preventive measures for a relatively longer period. For example, the United States updated guidance on mask wearing based on risk in February 2022 and canceled the order requiring negative COVID-19 test results before boarding a flight to the United States in June 2022 [53]; in contrast, Hong Kong rescinded similar preventive measures only in March 2023 and December 2022, respectively. Moreover, in Mainland China, the major shift in the zero-COVID policy in early December 2022 and a national omicron outbreak afterwards significantly influenced the economic and social lives of local residents [54], which may have affected the mental health of Mainland Chinese students studying in Hong Kong. Therefore, this study could contribute to the literature by investigating the challenges and coping strategies of university students at this unique location and timepoint.

Participants were sampled through purposeful sampling. All participants were undergraduate students at the university. This study was part of a large-scale mixed-methods study on university students’ mental health amid the COVID-19 pandemic [12,13,55]. Therefore, the recruitment methods included (1) sending invitations to participants in our quantitative study and (2) hiring student helpers to recruit targeted participants. Financial incentives were offered to students to encourage their participation. A short questionnaire was sent to the consenting students, and they were divided into different categories according to their responses. The following three categories, which had not been thoroughly examined in the scientific literature, were chosen as the focus of this study: (1) students with financial difficulties, (2) students from Mainland China and (3) students with a high level of self-perceived resilience (i.e., students who coped well). Around 20 students were recruited for each category, and each category of students was conveniently divided into three to four focus groups. It resulted in 56 participants divided into 10 focus groups. Supplementary Table S1 shows the three categories, related questions asked before the focus groups, the language used in the focus groups, the participant codes and the number of participants in each category.

Data saturation was achieved through consideration by the two authors. The number of participants in each category was determined with reference to the suggestions in the existing literature. Although researchers have reported insufficient and inconsistent standards for data saturation in focus group and thematic analysis [56,57], Malterud et al. [58] developed a model to systematically reflect various dimensions in a study that would impact its information power. This model argues that a study with a narrow study goal, a theoretical framework, high sample specificity, high-quality dialogue in the interviews and in-depth exploration of narratives would require a small sample size. Based on this theoretical model, we determined that a sample size of at least 15 for each category could offer sufficient information power.

Regarding the demographic information of the participants, they were Year 2 to Year 4 undergraduate students, with ages ranging from 19 to 25. Their major programs belonged to different schools, faculties and departments at the university, including the Faculty of Construction and Environment, the Faculty of Health and Social Sciences, the Department of Applied Biology and Chemical Technology, the Faculty of Engineering, the School of Nursing, the School of Fashion and Textiles, the Faculty of Humanities and the Faculty of Business. This study only collected the above demographic information and students’ self-reported background information in the short survey. No further information was collected to protect the privacy of the participants and encourage students’ active participation.

Written consent for data collection and recording the focus groups was sought from the participants. Students’ voluntary participation, measures for protecting their identities and data storage security measures were explained in the consent form. Students were assigned a code, and they were denoted by the code number throughout the focus groups to protect their personal identity. Because of the social distancing measures in Hong Kong, the focus groups were conducted via Zoom. These focus groups were video-recorded using the built-in function on Zoom. Each focus group was moderated by an experienced researcher and a research assistant and followed a semi-structured focus group guide (Appendix A.2.). During the focus groups, students took turns answering the moderators’ questions. The moderators asked follow-up questions based on students’ answers. The research assistants wrote brief notes during the focus groups for later analysis.

### 3.3. Data Analysis

The thematic analytic method was employed to analyze focus group data. Thematic analysis is frequently used in social science and psychology qualitative research to identify themes or patterns in qualitative data based on research questions [59]. Braun and Clarke [47] categorized thematic analysis into “coding reliability”, “codebook” and “reflexive” approaches. This study adopted the “codebook” approach, which was guided by a pre-determined theoretical framework or codebook. The “codebook” approach to thematic analysis merged “coding reliability” and “reflexive” approaches, adopting the philosophy of “reflexive” approaches and applying the structured approach to coding at the same time [60]. Moreover, researchers discussed different approaches for deductive qualitative research, and the template approach applied the codebook template to structural analysis [61,62]. It could facilitate theory testing and refinement by systematically and empirically examining pre-existing theories. The deductive approach to qualitative research could bridge research findings with existing theories and empirical findings. This enabled researchers to engage with previous theories, build upon other studies and develop a body of knowledge in the field [63]. The findings from this study could extend or refute the Transactional Theory of Stress and Coping, which has been widely used in the coping literature. The discussion and accumulation of results aimed to facilitate further explanatory research and intervention on coping.

This study employed the guidelines outlined by Fife and Gossner [21] and Naeem et al. [22] on deductive qualitative research. First, research questions were developed and a guiding theory was chosen. Second, the theory was operationalized using related scales. Third, research participants were sampled through purposive sampling. Fourth, after the focus groups, the brief notes written by the research assistants and the verbatim transcription of the focus group discussions were used for data analysis. The research assistants transcribed the audio recordings into text and translated Chinese transcripts into English. The authors double-checked the accuracy of the transcriptions and translation by listening to the recording again. Then, the authors familiarized themselves with the data and selected representative quotations from the transcriptions. Fifth, the authors selected keywords and coded the transcriptions through a deductive analytic approach using NVivo 15 software. Our deductive research adopted pre-existing theories to generate and categorize keywords and codes and develop themes from data. The codes were revised during the coding process based on regular discussion between the authors. Sixth, themes were developed by grouping codes in meaningful ways representative of the data. The last step involved conceptualizing the keywords, codes and themes [22]; interpreting evidence; and proposing confirmations and revisions of the guiding theory [21].

### 3.4. Rigor and Trustworthiness

This study upheld the principles of qualitative research proposed by Shek et al. [64], such as stating its philosophical basis, justifying the sample size, outlining the data collection procedure, highlighting the subjectivity and potential biases of the researchers through presenting their reflexivity statements, conducting inter-rater reliability testing to assess interpretation stability between researchers, ensuring the high auditability of the study by clearly explaining the decision trail of the study and providing additional illustrative quotes for each theme, checking alternative explanations of the findings and negative cases and documenting the limitations of the study. Furthermore, for qualitative research on psychology, the importance of methodological integrity was stressed [65,66]. This was achieved by examining the coherent function of subject fidelity and goal achievement utility in study design, data collection and data analysis.

## 4. Results

Data analysis based on the eight Ways of Coping Scales revealed that the participants used problem-focused coping (*n* = 24), emotion-focused coping (*n* = 46) and mixed problem/emotion-focused coping (*n* = 29) to cope with stress during the pandemic. With reference to the eight scales in the Ways of Coping Scales, seeking social support (*n* = 29), problem-focused coping (*n* = 24) and emphasizing the positive (*n* = 22) were most frequently applied by the research participants, followed by tension reduction (*n* = 17), distancing/detachment (*n* = 6) and self-isolation/keeping to themselves (*n* = 3). No participants applied wishful thinking and self-blame to cope with stress during the pandemic. Inter-rater reliability was assessed to ensure the stability of interpretations across the researchers. Twenty quotations from the focus groups were randomly selected and presented to two independent researchers. They were invited to re-code these quotations. The results revealed a satisfactory inter-rater reliability (percentage agreement on presence ≥ 70%) [52]. Table S2 summarizes the use of coping strategies by each participant in the eight Ways of Coping Scales and consolidates the results of this study. The following sections discuss the themes derived from the deductive analysis. For additional illustrative quotes regarding the themes, please see Supplementary Table S3.

### 4.1. Seeking Social Support, Emphasizing the Positive and Using Tension Reduction to Manage Negative Emotions

Research participants stressed the significant influence of negative emotions on their mental health during the pandemic; for example, many were depressed, anxious, frustrated, feared, worried, stressed, angry, annoyed, irritated and hopeless. These negative emotions stemmed from various challenges that they faced during the pandemic, including home confinement, travel restrictions and limited social activities. The fast-changing pandemic situation and the increasing number of infection cases in the city also caused significant anxiety among the research participants. This study found that seeking social support, including talking to their family members and friends about their feelings, was an important part of young people’s life during the pandemic. It helped to relieve them from the negative feelings that they experienced. Moreover, students preferred to share their feelings with parents or friends for different reasons. They would talk to their friends to seek reassurance from peers, but they would talk to their parents to seek advice, as the students explained:

*If I need to relieve stress, I would choose to talk to my friends because I feel that friends are peers, and they may have experienced things that are similar to me. So, I think I may be looking for reassurance from peers. When I share my problems with friends, I feel that they can give me viable suggestions. If I talk to my parents, they may have a perspective from the previous generation, and their views may be different from mine. So, I think that talking to friends helps me to relieve my negative feelings, and it may be better than sharing my feelings with my family members.*

(Student with financial difficulties, Group B, F-B-2).

*When I have some worries, I usually talk to my mom. She would share her experiences and perspectives, which help me solve some problems. When I was struggling with my studies, she told me to put less pressure on myself and to relax a bit.*

(Student with financial difficulties, Group B, F-B-6).

Furthermore, students could only have limited face-to-face interactions with friends during the pandemic. They felt isolated, which caused significant mental distress. Thus, research participants relied on video calls and phone calls with friends and family members. This communication channel was especially important for Mainland Chinese students studying in Hong Kong, who had family members and friends staying in Mainland China, as a student described:

*I just feel that family and friends are very important in the process. Because I was basically alone in the quarantine, chatting with my family and friends was the only way to mitigate my anxiety in the loop of assignments and online lessons.*

(Mainland Chinese student, Group B, M-B-9).

Additionally, students were confined to their homes during the pandemic, which significantly changed their living habits. This negatively influenced the physical and mental health of some students. For students who had mental illness before the pandemic, the pandemic further deteriorated their mental health, and some of them needed to seek professional help, as a student stated:

*I think the change of living habits has really destroyed my motivation. I think the influence still exists now. During the lockdown, I watched videos all the time, so my abilities to think deeply and concentrate for a long time need to be reconstructed. My way of thinking is really disordered now. I have gone for mental health consultation continuously during the pandemic. Because of my previous mental illness and my situation during the pandemic, my mom advised me to go for consultation. I have gone for consultation for two to three months.*

(Mainland Chinese student, Group A, M-A-8).

Another strategy applied by the research participants to release negative emotions was emphasizing the positive by doing something creative. They adjusted their mindsets and did something interesting to cheer themselves up. For example, although students’ travel plans were canceled due to the pandemic, they found ways to discover interesting places to visit in Hong Kong, as a student stated:

*Although the pandemic restricted my exchange plans, I still managed to discover new aspects of Hong Kong and looked for other joys in life with an open mind. Even though my original plans did not come true, I tried to find new opportunities and fun. Even though I could not travel, I still discovered interesting local things. I learned to take different approaches during the pandemic and still explored the world.*

(Student who coped well, Group B, C-B-2).

Furthermore, home confinement during the pandemic caused loneliness in young people. When students needed to undergo a 14-day home quarantine period due to COVID-19 infection, they experienced more negative emotions and a loss of connection with the nature. The research participants tried to find interesting things to do at home, for example, “developing hobbies such as reading, crafting, and cooking” (Student who coped well, Group A, C-A-4). These activities enabled them to release their negative emotions, such as loneliness and depression and cheer themselves up. Participants also tried to improve their mood at home by “taking care of [their own] appearance” (Student with financial difficulties, Group C, F-C-8) and “tidying the house” (Student with financial difficulties, Group C, F-C-9). These self-care activities allowed them to maintain healthy living habits during the pandemic.

Moreover, because of prolonged periods staying at home, young people may have experienced a lack of physical exercise, which would deteriorate their physical and mental well-being. Students also worried that poor physical well-being may increase their risk of contracting COVID-19, which could negatively affect their academic performance. Various tension-reduction strategies were employed by research participants to resolve related stress, for example, performing physical exercise and getting away from their problems for a while. Research participants highlighted the effectiveness of performing physical exercise to reduce their negative emotions, as a student explained: “I began to do sports and I felt really good after performing physical exercise. It could reduce stress” (Student with financial difficulties, Group A, F-A-2). Moreover, visiting the nature could help students to reduce the feeling of being trapped at home during online learning, as a student stated:

*My way to relieve stress is to go to the nature and breathe fresh air. As I said before, I feel very stuffy at home, even if my family members stay with me, I don’t feel good. Sometimes I go hiking with my friends or family members. The most frequent place I visit is a place called ‘Tai Long Wan’ in ‘Chai Wan’, where we could hike for two to three hours and eat and play in the water all day.*

(Student with financial difficulties, Group C, F-C-5).

However, because of the health risks related to going out during the pandemic, some research participants chose to perform home workout activities, such as meditation and breathing exercises, which were also effective at reducing their negative emotions, as a student explained:

*It is mainly about stress and anxiety. I tried some breathing exercises. It was an instant stress reduction, and my mood was much calmer. Because I was confined to my home, my options were limited. So, I had to explore things that were available on the Internet, and I came across breathing exercises.*

(Student with financial difficulties, Group A, F-A-4).

Furthermore, this study found that most students with personal or family financial difficulties chose to perform physical exercise to release their negative emotions. Out of the eleven students who employed this strategy, ten were students with financial difficulties. One of the possible explanations was that students with financial difficulties in Hong Kong usually had smaller living spaces at home, so physical exercise released the feeling of being trapped at home for these students.

### 4.2. Problem-Focused Coping and Emphasizing the Positive to Deal with Health-Related Anxiety and Stress Arising from the Frequent Change in Pandemic-Related Policies

Research participants had a high level of anxiety because of various health risk related to COVID-19, including feeling worried at the beginning of the pandemic because of the limited understanding of this disease and being afraid that the older people in their families would have serious health risks from infection. Thus, problem-focused coping strategies were applied when facing the uncertainty related to COVID-19 infection and applying effective preventive measures. They tried to gather more “reliable information sources [related to the virus], prevent being misled by rumors” (Student who coped well, Group B, C-B-7) and be more rational when taking pandemic-related preventive measures. One student said that being rational was important for him to avoid experiencing extreme negative emotion when managing COVID-19 infection risk, describing his experience as follows:

*Sometimes I fell into a deep panic and fear, and it was very immersive. For example, I thought I had contracted COVID-19, and I thought about it all the time. However, in reality, I might not have contracted COVID-19. Moreover, when I coughed, I would ask myself whether I have a fever, and whether I had been to high-risk area. If my chance of being infected was low, I would try not to drown into anxiety.*

(Student with financial difficulties, Group C, F-C-8).

Moreover, family members contracting COVID-19 caused significant mental pressure on the research participants, and they would apply emphasizing the positive as a coping strategy. They tried to be more optimistic about the future, as a student described:

*I was very worried after my family members were diagnosed, and I searched everywhere for home remedies or ways to [help them] recover quickly. However, at the end, I told my family members and myself to wait patiently and we would eventually recover. It was actually not as serious as I imagined.*

(Student who coped well, Group C, C-C-4).

Furthermore, the Hong Kong government implemented various preventive policies to control the spread of the disease and protect the health of citizens, including the mask order, travel restrictions, restricted dine-in hours in restaurants, quarantine measures for infected persons and travelers and vaccine pass requirements. The frequent change in policies created confusion and uncertainty, especially in older people, making it challenging for them to adapt and adjust effectively. Young people also found that the preventive policies were complicated, which made them question their necessity. Some policies instilled fear in the community and led to panic-driven behaviors in citizens (e.g., panic-buying). Young people were also afraid that older people in their families would blindly believe the information presented on the television or the Internet. To cope with the stress caused by pandemic-related policies, research participants applied the coping strategy of emphasizing the positive, as a student stated:

*To handle the information explosion and my negative emotions, I think the most important things are maintaining discernment, adjusting my mindset, and holding on to hope. It is important to adjust my mentality. If I was overwhelmed by too much information, I would take a break and use my judgement to distinguish between useful and harmful information. It is also important to stay hopeful and positive. Even though we have a lot of unfinished business, we can still focus on other aspects, such as learning new skills and preparing for the future.*

(Student who coped well, Group B, C-B-6).

Furthermore, the Mainland Chinese government implemented policies that were different from those in Hong Kong. At the early to middle stages of the pandemic, the zero-COVID policy led to very strict preventive measures in Mainland China (e.g., the quarantine measures for travelers). This significantly affected students who needed to travel between Hong Kong and Mainland China during the pandemic, as stated by a student:

*At the end of January 2022, many of my friends thought that Hong Kong would have a big outbreak, so they went back home in Mainland China, and they needed to scramble for bus tickets. […] My trip to home was not easy. I went back two times, so I got quarantined four times, and the longest one was 21 days. The nucleic acid test requirement also bothered me.*

(Mainland Chinese student, Group A, M-A-5).

To cope with the stress and challenges related to these preventive policies in Mainland China, students also applied the coping strategy of emphasizing the positive, as a student stressed:

*Our mentality is the most important. Trying to make myself stay calm could release my anxious feelings. I treat the pandemic as a pause in my life. I keep thinking about what I could do to enrich my life at the moment. The things I didn’t have time to do before the pandemic are now ready to be done. I don’t pay attention to the pandemic, and I don’t take it too seriously.*

(Mainland Chinese student, Group C, M-C-4).

This study also found that students with high levels of self-perceived resilience (i.e., students who coped well) frequently applied the strategy of looking for a silver lining and on the bright side. Out of the nine students who employed this strategy, eight were students with a high level of self-perceived resilience during the pandemic. They tried to look on the positive side when facing difficulties and believed that the future would be better, as a student stated:

*It is easier for us to adapt to sudden changes and look for opportunities. Instead of dwelling on the negative, we should focus on the positive. We may be upset when events were cancelled, but we should believe that we could go through [the pandemic] together.*

(Student who coped well, Group B, C-B-4).

This finding demonstrated the significant relationship between young people’s positive reframing of adversity and their resilience qualities during the pandemic.

### 4.3. Seeking Social Support, Tension Reduction, Distancing/Detachment and Self-Isolation/Keeping to Themselves to Handle Conflict with Family Members

Conflict with family members was prominent among the research participants. Spending more time at home increased their chances of having conflict with family members. Conflict occurred when family members fought for space and resources at home, such as computers and televisions, or when there was a difference in lifestyle among family members. Students also needed to do more housework when staying at home longer, particularly cleaning, and conflict might occur over the distribution of housework among family members. Moreover, the pandemic led to more closeness among family members, but excessive care and attention could sometime cause conflict between them. They might quarrel over whether it was safe to go out of the house, getting vaccinated and practicing preventive measures. The findings showed the importance of peer support for reassurance and providing advice to resolve related problems, as a student stated:

*I would talk to my friends immediately when I have family conflict. My friends would say they undergo the same thing. It made me feel better to know that everyone was going through similar situations. When I talked to my friends, they would share their own experiences on how they resolved conflict with their family members.*

(Student with financial difficulties, Group B, F-B-8).

Moreover, family members quarreled more with each other because of the negative emotions accumulated during the pandemic (e.g., anxiety, worry, blame). They needed a channel to vent, which led to more arguments. Sometimes, the situation became so serious that the students needed to seek professional help to deal with family conflict, as a student described for her situation during the pandemic:

*The main problem I faced was the conflict within the family, which led to my psychological imbalance. After seeking help from a counsellor, I found that it has helped me to deal with the difficulties.*

(Student who coped well, Group B, C-B-5).

Furthermore, family conflict could arise from the family’s financial difficulties during the pandemic. All members in a family needed to share the financial burden, and young people needed to balance school and work. Family members could quarrel when discussing financial issues or become impatient or nervous about the family’s financial situation, leading to more arguments. The research participants applied a mixture of different coping strategies (tension reduction and seeking social support) to handle stress arising from family conflict, as a student stated:

*Don’t stay at home for too long. Don’t spend too much time at home. [Staying at home for too long] can lead to more accumulated negative emotions and more conflict with family members. Going out more often with friends can help to resolve the problems. We should also be tolerant of our family members.*

(Student who coped well, Group A, C-A-2).

Some students applied the coping strategy of distancing/detachment to handle conflict with family members. Although researchers usually regard it as a more passive way of coping, findings from this study showed that it could be effective for young people to reduce the stress caused by family conflict, as a student described:

*When I was really angry [with my family members], I would relax by listening to music or watching the variety shows that I like. I would put on my headphones and just ignore them. I would not listen to them or I would just pretend I haven’t heard anything. That’s it.*

(Student with financial difficulties, Group B, F-B-7).

Although students from Hong Kong had more conflict with family members because of increased contact with them, the story was different for Mainland Chinese students who did not return home during the pandemic. Some of them missed family gatherings for a few years because of college entrance exams and the pandemic. They really missed their family members in Mainland China and would often call them or send messages to reduce their homesickness. Students also applied the coping strategy of self-isolation/keeping to themselves to handle the negative emotions resulting from homesickness, as a student stated:

*Before the pandemic, I used to ask for help from my mom. However, during the pandemic, I prefer to digest the homesick feeling by myself. I thought that calling my mom would make my homesickness worse. I would handle it by myself and would only call her sometimes.*

(Mainland Chinese student, Group C, M-C-5).

Moreover, towards the end of 2022, when the Mainland Chinese government lifted the zero-COVID policy, many people were infected with the disease. Mainland Chinese students studying in Hong Kong were worried that their family members and friends in Mainland China would have significant health risks, so they tried to reduce related stress by maintaining close communication, as a student described:

*Recently, because of the outbreak of the pandemic in Mainland China, all my family members were infected, which have caused me great concern. […] I would contact my family members more often and spend less time alone. Now, I try to communicate with people as much as possible and cherish face-to-face meeting opportunities, which can relieve my anxiety.*

(Mainland Chinese student, Group B, M-B-4).

### 4.4. Seeking Social Support, Problem-Focused Coping, Emphasizing the Positive and Using Distancing/Detachment to Cope with Challenges in Online Learning and Academic and Career Development

Problem-focused coping was used by the research participants to resolve online learning challenges. To enhance their physical and mental well-being in online learning, students tried to maintain a normal daily routine. Moreover, online learning required different learning skills from students. They often found it difficult to concentrate in online lectures or did not have a good study environment at home or in their dorm room, so they needed more self-discipline and effective time management. Some students thought that online learning did not provide hands-on practice opportunities for them, so they could not acquire practical skills necessary for their professions. Furthermore, they could not have face-to-face interactions with teachers and other students through online learning. Although students were assigned to break-out rooms during online lectures, they generally did not participate actively in the online discussion. Some also found it more difficult to ask questions during online lectures, or the professors did not fully utilize the features in the online learning platforms, leading to inefficient learning. Students applied problem-focused coping by finding different ways to resolve related problems, as a student described:

*There may be times in the learning process when a professor did not follow school policies, such as not videotaping the lectures or not allowing students to view the lecture recordings. Students may complain about the situations, but the department chair may not be able to do anything about it. When facing such difficulty, students were left to find other resources to supplement their learning. For the knowledge we cannot fully comprehend in online lectures, we would need to learn it by ourselves. Overall, my learning process [during the pandemic] was characterized by a variety of challenges that needed to be resolved by taking initiatives myself.*

(Student who coped well, Group A, C-A-1).

Many research participants also sought support from friends to deal with online learning challenges. As they made friends with colleagues at the university, they could study together and supervise each other. This kind of peer support helped them to cope with various challenges in online learning, as a student stated:

*My friends helped me when I was studying at home and cannot concentrate well. When I had a short deadline or needed to submit my project very soon, especially during the exam period, I might invite some friends to join me on Zoom or Teams to study together. When we were bored, we may watch a couple episodes of cartoon together.*

(Student with financial difficulties, Group B, F-B-6).

Furthermore, students faced a high level of uncertainty about their academic and career development. Because of the reduction in hands-on practice and laboratory exercise for some university majors, students felt less prepared for their future academic and career development. Some departments reduced the content of certain courses, so students learned less. Some professors organized open-book instead of close-book exams for students, which may not have been effective at assessing students’ performance. Moreover, some students could not attend internships during the pandemic and their graduations were delayed. Some students needed to work from home for their internships, which limited their chance to learn more practical skills, communicate with real clients or patients and ask for help from colleagues. These difficulties during the pandemic reduced students’ confidence in their future academic and career development, as a student stressed:

*Although the Hong Kong and Mainland Chinese governments are not applying strict lockdown measures at the moment, my previous experience has traumatized me. I am not sure how the future will turn out and whether I could complete my 4-year university study. I feel insecure and I am not able to think about my future. I also feel that time flies and I am still confused about my future.*

(Mainland Chinese student, Group A, M-A-2).

Additionally, the pandemic brought a lot of uncertainty to the economy and different industries, which increased students’ stress about their future career development. Some companies laid off workers in the early to middle stages of the pandemic, which made students consider changing their career goals. However, in the later stages of the pandemic, some industries recovered and some university departments held recruitment talks for students every week, which made students less worried about their future careers. The findings showed that university students’ career development was significantly affected by the social and economic environment. Students coped with related stress by applying the coping strategy of emphasizing the positive, which helped them stay motivated even during difficult times, as a student stated:

*The most difficult thing was that, after being diagnosed, I couldn’t go to work and felt very anxious. There was no response from my job applications. Fortunately, I found a new job after a while. The most important thing is not to give up and reduce my negative emotions. Then the path for our boat will straighten naturally when it reaches the bridge.*

(Student who coped well, Group D, C-D-1).

Moreover, when students found that there was no immediate solution to their problems, they employed the coping strategy of distancing/detachment, which could act as a kind of escape for them, and they would feel better and gain motivation after a short period of escape from the situation, as a student explained:

*A more passive coping method is to ‘let it go’. When I really couldn’t stand it, I would let it go. I just did nothing in bed all day. Watching TV dramas may help sometimes. Because I am an introvert, I can charge myself when I am alone. When I let it go, I would force my brain to shut down, and then I am in a state of escape. After a short period of escape, I feel like I can gather some courage and motivation to continue moving forward. Although the situation might be bad at the time, after a short escape, I would be willing to make some changes.*

(Mainland Chinese student, Group A, M-A-2).

Furthermore, the pandemic decreased students’ opportunities for face-to-face social interaction, meeting new friends and expanding their social circles at university. Some students started their university life when the pandemic began, so they had only attended online lessons at university, which made them feel like they did not have a holistic university experience. This negatively impacted both students’ careers and personal development, as a student explained:

*I have met fewer people, but actually, these people perhaps can help me get some very important information. After I get this information, maybe my career planning or post-study planning will become clear. However, since we were all taking online classes in the first two years of the pandemic and I was not very good at socialization, I knew very few people. The information I am getting now is probably limited compared to other students. I could only get some more valuable information until the second year at the university, and I feel that I am already left behind.*

(Mainland Chinese student, Group A, M-A-7).

Students tried to resolve this problem by employing problem-focused coping, as a student stated:

*My challenge is the lack of social interaction. I have a slow-to-warm-up temperament, so the solution is to take the initiative to contact my old friends and make new ones. Now that we are starting to recover from the pandemic. I will look for opportunities to meet more people and build social relationships.*

(Student who coped well, Group D, C-D-4).

The findings of the current study showed the problems related to online learning and academic and career development faced by university students during the pandemic. Students tried to resolve these problems by applying various coping strategies, including seeking social support, problem-focused coping, and emphasizing the positive and distancing/detachment.

## 5. Discussion

This study found that problem-focused coping, emotion-focused coping and mixed problem/emotion-focused coping were all used by university students to cope with their stress during the pandemic. Among the eight Ways of Coping scales, seeking social support, problem-focused coping and emphasizing the positive were most frequently applied by the research participants. The literature shows that university students in different countries applied different coping strategies. Investigating Pakistani undergraduate students in the early stage of the pandemic (i.e., May 2020), Bana and Sarfaraz [67] found that the most frequently applied coping strategies were meditation, taking breaks from watching or reading COVID-19-related news and distracting themselves with other activities. Examining Polish university students in November 2020, Guszkowska and Dąbrowska-Zimakowska [68] found that the most frequently employed coping strategies were accepting the situation, active coping, distracting themselves with other activities and performing physical activity. Surveying Mainland Chinese college students from February to October 2020, Liu et al. [69] found that they employed more active coping styles (23.9%) than negative coping styles (17.4%). Another study on Mainland Chinese college students in October 2022, which was after the prolonged campus lockdown, found that 62.6% of students spent their free time surfing the internet, which was regarded as a negative coping strategy [70]. In late 2022 to early 2023, when Mainland China was experiencing the national omicron outbreak, Zhao et al. [71] showed that Mainland Chinese adults’ mental health and risk-related information sharing (which was regarded as a form of coping behavior) were better than in the initial phase of the pandemic. These studies showed the significant differences in individuals’ choices of coping strategies based on their country of residence and the stages of the pandemic. However, qualitative studies on Hong Kong university students were not found, especially during the later stage of the pandemic, so the current study contributes to the literature by demonstrating their coping attitude and behaviors at this unique location and timepoint.

This study found that students with a high level of self-perceived resilience employed the coping strategy of emphasizing the positive more frequently than students with financial difficulties and Mainland Chinese students studying in Hong Kong. Studies conducted during the pandemic also showed the significant relationship between young people’s resilience and their positive reframing or reappraisal of life challenges during the pandemic. For example, positive and optimistic thoughts had significant influence on Indian university students’ resilience during the COVID-19 pandemic [72]. Mindfulness, optimism and resilience were found to moderate the relationship between COVID-19 fear and mental distress in Dutch and Belgian adults [73]. This study demonstrated the significant relationship between young people’s positive reframing of adversity and their resilience during the pandemic.

Another coping strategy, tension reduction, was most frequently applied by students with financial difficulties. Researchers studied the use of coping strategies by individuals with financial difficulties during the pandemic, but most of the studies were conducted on general adults. Still, mixed results were found: the perception of financial threat could lead to more problem-focused coping strategies [74], another study found that low financial status led to a low level of confidence in individuals’ ability to cope and less use of active coping strategies [75] and another study on Brazilian undergraduate students with lower family incomes showed that they were more likely to use positive re-evaluation strategies [9]. This study showed more frequent use of tension reduction by university students with financial difficulties during the pandemic, which could have been caused by the small living spaces of students with financial difficulties in Hong Kong.

### 5.1. Coping with Accumulated Negative Emotions

This study found that university students employed the coping strategies of seeking social support, emphasizing the positive and tension reduction to manage negative emotions. Peer support and connecting with family and friends through digital devices were especially helpful for Mainland Chinese students studying in Hong Kong. Studies conducted during the pandemic showed the positive benefits of social support to university students’ mental health. It could act as a protective factor against mental distress and loneliness [76] and increase their life satisfaction and positive feelings [77]. Researchers also found that international university students were more likely to suffer from deteriorated mental health, and there were negative relationships between social support and mental distress [78].

Additionally, this study showed that, because of the lockdown measures and online learning arrangements, many academic and non-academic activities were canceled or postponed, so university students were confined to their homes during the pandemic. To cope with the negative emotions resulting from the confinement, participants entertained themselves with creative activities. The literature shows similar findings: college students would reduce their negative feelings by distracting themselves with creative activities, learning new technical skills [79], employing positive reframing and maintaining regular daily routines to cope with academic pressure and restricted social encounters [80].

Moreover, this study found that performing physical exercise could release research participants’ stress and negative emotions. Going out of the house or visiting nature could reduce negative emotions related to being trapped at home. Another study also revealed that physical activities could effectively reduce Chinese college students’ negative emotions [81]. Furthermore, researchers showed the importance of being in nature to individuals’ mental health during the pandemic: Chen and Ye [82] found that Chinese university students’ time spent in nature or outdoor activities was positively related to their subjective well-being during the pandemic. Findings from this study align with the literature on the effectiveness of physical activities and visiting nature for coping with stress during the pandemic.

### 5.2. Coping with Health-Related Anxiety and Stress Arising from the Frequent Change in Pandemic-Related Policies

In the literature, studies also found that young people faced significant stress from the health risk and pandemic-related policies. A survey of university students in Israel found that 64.4% worried for their family members’ health [83]. Another study found that a high percentage of German university students (75–78%) feared someone from their personal network or close to them becoming infected with COVID-19 or being severely ill [84]. Moreover, a survey of college students in seven countries or regions found that students were concerned about the impacts of politics, the economy and misinformation on society [85]. Examining US undergraduate students from March to May 2020, Stamatis et al. [86] showed that “disruption due to the pandemic” and “limited confidence in the federal government’s response” significantly predicted their depression symptoms after controlling for baseline depression.

This study found that university students applied problem-focused coping and emphasizing the positive to cope with health-related anxiety and stress arising from the frequent change in pandemic-related policies. The results from this study aligned with findings from the literature. A study showed that understanding the risk of COVID-19, knowledge of its prognosis, preventive measures and wearing face masks protected college students against their COVID-19 fear and mental distress during the pandemic [87]. Another study of Pakistani university students found that students having someone in their personal network being infected with COVID-19 employed more acceptance coping [88]. This study demonstrated the importance of problem-focused coping and emphasizing the positive to university students in Hong Kong for coping with health-related anxiety and the frequent change in pandemic-related policies.

### 5.3. Coping with Family Conflict

The literature also shows that, due to spending more time with family members, young people may have had more conflict with them. Vu et al. [89] revealed that the social separation policy in Vietnam during the pandemic led to more parent–adolescent conflict. Individuals’ responsibilities and privileges at home were among issues that caused disagreements. Another study highlighted that parental conflict was an important risk factor for adolescents’ adjustment to the pandemic [90].

This study found that university students applied the coping strategies of seeking social support, tension reduction, distancing/detachment and self-isolation/keeping to themselves to reduce related stress. Aligning with the findings from this study, prior studies showed that social support and tension reduction were important for helping young people to cope with stress resulting from family conflict. Liu et al. [69] examined Chinese college students’ family functioning and mental health problems during the outbreak, remission, online study and school reopening phases in 2020. The study found that family dysfunction and the use of a negative coping style worsened college students’ depression and anxiety, while people with an active coping style could create a positive feedback mechanism between active coping with family issues and better family functioning. Moreover, Ojewale [91] examined undergraduate students in Nigeria and found that negative family functioning was significantly associated with depression. Using the social media platform “WhatsApp” and watching television or movies were among the coping methods most frequently applied by the students. Studying Spanish college students during lockdown, Padrón et al. [80] found that interpersonal conflicts, including intensified family conflict, caused significant mental distress in students. The study found that positive reframing skills and maintaining daily routines were the most effective coping strategies.

Furthermore, this study found that some students applied the coping strategy of distancing/detachment to cope with stress related to family conflict. In the literature, studies also found that returning home for quarantine could be challenging for some college students. A study by Dotson et al. [92] studied US college students and found that many students lived with at least one parent during the sheltering-in-place order. They reported increased conflicts with family members over pandemic-related precautionary measures and emotional distancing with peers resulting from the loss of affective connections. This created “developmental mismatch” in young people because emerging adulthood is usually characterized by developing new friendships and romantic relationships independently rather than being restricted in isolated environments with family members. Findings from the current study complemented the existing literature by showing the use of distancing/detachment by young people to cope with stress related to family conflict in this phase of “developmental mismatch”.

Moreover, this study revealed the use of the coping strategy self-isolation/keeping to themselves by young people to cope with family conflict. In the literature, researchers stated that parent-related loneliness in adolescents is considered a “normal” dimension of loneliness experience that increases their distance from parents [93], while peer-related loneliness is related to mental distress [94]. During the COVID-19 pandemic, there were limited studies on the positive benefits of self-isolation for young people. Thus, this study filled this knowledge gap by demonstrating how the pandemic provided an opportunity for university students to gain independence by increasing their distance from their parents.

### 5.4. Coping with Challenges in Online Learning and Academic and Career Development

In the literature, researchers found that young people faced online learning challenges and significant uncertainty during the pandemic, including the loss of structure and resources in their university lives, the interruption of their academic timelines and negative identification with their potential future career [95]. Studying university students in Israel, Schiff et al. [83] found that 63.2% experienced online learning difficulties. A study of UK university graduates showed that the pandemic increased graduates’ concerns about their job opportunities and employer support. Graduates also worried that the pandemic may harm the labor market and their future career prospect [96]. A study of Italian university students revealed their high levels of uncertainty about employability and career planning during the pandemic [97].

This study found that research participants applied the coping strategies of seeking social support, problem-focused coping, emphasizing the positive and distancing/detachment to cope with challenges in online learning and academic and career development. First, literature showed that college students applied distancing/detachment and emphasizing the positive to cope with uncertainty. Studying medical students in the US, Kerr et al. [95] showed that distraction, avoidance and positive reframing could facilitate students’ coping and management of uncertainty. Second, the literature revealed that university students could benefit from seeking peer support during online learning. A study of engineering college students during the pandemic showed that peer support in both remote and in-person settings was important for students in online learning [98]. 

Third, for problem-focused coping, studies showed its prevalence among university students and its effectiveness for coping with stress related to students’ learning and future development. Studying US college students, Von Keyserlingk et al. [99] revealed a significant increase in their level of academic stress amid the pandemic, while students’ self-efficacy in self-regulation predicted lower stress increases. For uncertainty about future development, researchers investigated the concept of “intolerance of uncertainty” and university graduates’ employment anxiety during the pandemic. Chen and Zeng [100] showed that intolerance of uncertainty positively predicted college graduates’ employment anxiety, moderated by career planning, highlighting the importance of career planning for helping college graduates to handle uncertainties brought by the pandemic. These studies demonstrated the important role of problem-focused coping in reducing stress related to individuals’ learning and future development during the pandemic.

### 5.5. Discussion on Coping Theories

This study’s findings could support and extend the Transactional Theory of Stress and Coping. In this section, we compare our research results with other coping theories proposed by researchers, including the Brief COPE Inventory, Kidcope, the Simplified Coping Style Questionnaire (SCSQ), the Multidimensional Coping Inventory (MCI), the Brief Approach/Avoidance Coping Questionnaire (BACQ) and the Responses to Stress Questionnaire (RSQ).

First, regarding seeking social support, this study illustrated the importance of this coping strategy for helping young people to cope with various challenges during the pandemic, including accumulated negative emotions, conflict with family members and challenges in online learning and academic and career development. Similar to the Transactional Theory of Stress and Coping, this coping dimension is highlighted in various existing theories on coping, including the Brief COPE Inventory (using instrumental support), the SCSQ (talk to people and confide your inner troubles), the MCI (spend time with a special person), the BACQ (talk to people when it gets to be too much) and the RSQ (emotional expression). However, some strategies related to seeking social support have been regarded as maladaptive or passive coping by some researchers. For example, venting is regarded as maladaptive in the Brief COPE Inventory and relying on others to solve the problem is classified as passive coping in the SCSQ. Our study found similar results. For example, when handling family conflict, a research participant thought that talking to friends about the problem “felt like I was escaping reality instead of facing the challenges” (Student who coped well, Group D, C-D-2). But some students thought that talking to friends about feelings could release accumulated negative emotions. Thus, future research could further investigate the conducive and undesirable effect of seeking social support on individuals to determine its adaptive or maladaptive nature.

Second, regarding emphasizing the positive, this study showed the importance of this coping strategy for helping young people to cope with accumulated negative emotions, health-related anxiety, stress arising from the frequent changes in pandemic-related policies, challenges in online learning and academic and career development uncertainty. This coping strategy is listed in the Brief COPE Inventory (positive reframing), Kidcope (cognitive restructuring), the SCSQ (try to see the good side of things), the BACQ (something positive could come out of problems) and the RSQ (cognitive restructuring and positive thinking). Moreover, under emphasizing the positive, engaging in creative activities is listed, and this coping strategy is also highlighted in other coping theories, including the SCSQ (seek hobbies and actively participate in cultural and sports activities) and the MCI (treat myself to a favorite food or snack and see a movie). This comparison shows that emphasizing the positive is a commonly found coping strategy in the coping theory literature.

Third, for tension reduction, this study demonstrated the importance of this coping strategy for helping young people to release negative emotions and handle family conflict. The Way of Coping Scale includes engagement in different activities (e.g., getting away, eating, exercising) to reduce tension resulting from the stressful situation. The concept of distraction is highlighted in various coping theories. It is mentioned in Kidcope (distraction), the SCSQ (get relief through work, study or some other activities), the MCI (take time off and get away from the situation), the BACQ (put problems behind, concentrate on something else) and the RSQ (distraction). However, distraction is regarded as a form of maladaptive (e.g., self-distraction and substance use in the Brief COPE Inventory), passive coping (e.g., trying to rest or take a vacation to temporarily put aside the problem or trouble in the SCSQ) or avoidance-oriented coping (e.g., take time off and get away from the situation in MCI) in other coping theories. Previous empirical studies have linked avoidance-oriented coping and passive coping with physical and mental distress [101,102] and low self-esteem [103]. Further investigation is needed to examine the benefit and harm of applying tension reduction as a coping strategy. Moreover, under tension reduction, engaging in physical activities is included, which is also listed in other coping theories, including the SCSQ (seek hobbies and actively participate in cultural and sports activities) and the BACQ (physical exercise is important). This study highlighted the effectiveness of physical exercise for improving young people’s mental health during the pandemic.

Fourth, for problem-focused coping, this study demonstrated the effective application of this strategy by young people for dealing with health-related anxiety and frequent changes in pandemic-related policies, and challenges in online learning and academic and career development uncertainty. The Ways of Coping Scale comprises various forms of problem-solving, including producing different solutions to problems, making plans of action, trying not to act too hastily, etc. These coping strategies are highlighted in various coping theories. It is named active coping and planning in the Brief COPE Inventory, problem solving in Kidcope, active coping (e.g., stand firm and fight for what you want) in the SCSQ, task-oriented coping in the MCI, active effort to find solution in the BACQ and problem solving in the RSQ. This comparison demonstrates that problem-focused coping is a commonly found coping strategy in the coping theory literature.

Fifth, distancing/detachment refers to the acceptance of the situation, trying to forget the problem and believing that time will make a difference in the Ways of Coping Scale. This study showed that university students applied this coping strategy to handle conflict with family members and to cope with challenges in online learning and academic and career development uncertainty. This coping strategy enabled them to release stress when the problem could not be resolved in the short term. This coping strategy is also highlighted in other coping theories, including the Brief COPE Inventory (acceptance), Kidcope (resignation), the SCSQ (believing that time will change the status quo and the only thing to do is to wait), the BACQ (try to forget problems) and the RSQ (acceptance). However, this coping strategy is regarded as negative coping in Kidcope and passive coping in the SCSQ. Previous empirical studies revealed the harmful effects of negative and passive coping on individual well-being [104] and youth violence [105], so more investigation is needed to further understand the positive and negative effects of distancing/detachment as a coping strategy.

Sixth, self-isolation/keeping to themselves refers to refraining from sharing feelings and situations with others and avoiding staying with people in general in the Ways of Coping Scale. This study demonstrated that this coping strategy could be beneficial for helping university students to gain independence by increasing their distance from their parents. This coping strategy is included in other coping theories, including the BACQ (withdraw from other people) and the RSQ (avoidance). However, this coping strategy can be regarded as either positive or negative in Kidcope (emotional regulation vs. social withdrawal) and as either active or passive in the SCSQ (try to restrain your disappointment, regret, sadness and anger vs. comforting oneself). This comparison reveals the various dimensions of emotional regulation. It could include suppressing negative feelings, comforting oneself and/or withdrawing from social interactions. Future research could further investigate or classify these dimensions to facilitate more comprehensive analysis of related coping strategies.

For the remaining two scales (i.e., wishful thinking and self-blame) in the eight Ways of Coping Scales, this study found no related results. However, the scales are highlighted in other coping theories, including the Brief COPE Inventory (self-blame), Kidcope (criticizing self and wishful thinking), the SCSQ (fantasizing that some miracle may happen to change the status quo), the MCI (blame myself for procrastinating or being too emotional about the situation, daydream about a better time or place, fantasize about how things might turn out) and the RSQ (wishful thinking). It shows that these coping strategies may be prevalent in other situations. Moreover, other coping theories contain coping dimensions that are not included in the Transactional Theory of Stress and Coping, for example, denial in the Brief COPE Inventory and the RSQ, blaming others in Kidcope and being on the way towards giving up in the BACQ. Future research could further investigate and compare these coping dimensions to enhance knowledge accumulation in the field.

### 5.6. Theoretical Implications

The findings of this study have important theoretical implications. First, this study supports and extends the Transactional Theory of Stress and Coping. Researchers have highlighted the lack of consensus about core coping categories, which created difficulties in accumulating results and knowledge for further explanatory research and intervention [27]. This study also contributes to the literature by analyzing the empirical findings based on the Transactional Theory of Stress and Coping and comparing the research findings with various coping theories. It reinforces the importance of coping mechanisms in human functioning. Since coping is a learned behavior shaped by previous traumatic experiences, understanding different reactions to stress helps us to develop healthy coping strategies [106]. Second, this study captured the unique challenges and coping strategies of Hong Kong university students during the pandemic. Using a qualitative research approach illustrated the contextual features of the city and the people, including preventive policies, the severity of the pandemic and the lifestyles of young people. As most existing studies were conducted in societies with WEIRD (Western, educated, industrial, rich and democratic) attributes, findings on Chinese participants could fill in to this knowledge gap. Third, data collection in this study was conducted at a unique time point (late December 2022 to mid-January 2023). Although many countries or regions had already loosened pandemic restrictions by this point, Hong Kong still had many preventive measures at the time, including a quarantine order for COVID-19 patients, travel restrictions between Hong Kong and Mainland China and a mask order. Thus, this study contributes to the literature by providing the retrospective view of young people’s challenges and use of coping strategies after prolonged pandemic restrictions. Moreover, Mainland China underwent a major shift in zero-COVID policy and a national omicron outbreak in early December 2022. This study examined the challenges and coping strategies of Mainland Chinese students studying in Hong Kong at this unique timepoint. Fourth, it specifically examined university students in three categories (i.e., students who coped well, Mainland Chinese students studying in Hong Kong and students with financial difficulties), which were not sufficiently examined in the literature, so this study filled this knowledge gap. Moreover, as there are few studies on the coping of emerging adults during the pandemic, social scientists can develop novel models based on the present findings and an ecological perspective [107].

### 5.7. Practical Applications

Regarding practical implications, this study identified various challenges faced by university students during the pandemic, which would help with planning effective interventions in the post-pandemic period. Employment and mental health issues still affect young people. Specifically, evidence shows that the pandemic increased young people’s suicide risk [108], and the long-term impact of loneliness on their mental health was not fully examined [109]. There has been an uneven recovery in the labor market in countries across the post-pandemic world [110]. Thus, this study provides evidence on the challenges and coping strategies of university students during the pandemic, which could facilitate the design of related policies and interventions in the future. Additionally, as another pandemic will likely occur in the future, the present findings provide pointers for preventive measures. Obviously, it would be helpful to enhance adaptive coping mechanisms in university students.

There are several recommendations to help higher education institutions in the context of a pandemic. First, healthy coping education should be included in the formal curriculum such as leadership subjects [111,112,113]. This could ensure that no student is left behind. Second, informal education programs can be conducted by student affairs offices to nurture the healthy coping responses of university students. Moreover, university students should be encouraged to form student associations on mental health to promote healthy coping. Finally, developing validated rapid assessment coping instruments using advanced statistical analyses [114] would be helpful for assessing coping in an objective and systematic manner.

### 5.8. Limitations

Several limitations of this study should be noted. First, the findings may only explain the stress and coping strategies of the research participants sampled in this study. Findings may be generalized to other university students based on the naturalistic generalizability principle of qualitative study [115]. This concept emphasizes readers’ own determination stemming from their related experiences and understanding of the phenomenon in this study [116]. Second, this study only investigated participants’ retrospective views of their stress and coping strategies in the three-year pandemic in Hong Kong, so we may not fully understand their related changes over time. Future studies may apply a longitudinal study design to examine the changes in coping strategies over a period after the pandemic. Third, the themes developed in this study were presented based on the challenges faced by university students (e.g., negative emotions, conflict with family members), but potential interactions between themes should be considered when interpretating the findings. For example, the inter-relationships between conceptions of coping and ego orientation involved in coping should be further investigated [117]. Fourth, this study mainly determined data saturation based on an existing theoretical model. The method for determining data saturation could be further improved by calculating the base information size, run length and new information threshold when conducting the focus groups [118]. Finally, as a deductive approach was adopted, this study may not have identified themes not covered in the proposed theoretical framework [119]. Future studies may adopt an inductive approach to qualitative study to explore new categories of coping.

## 6. Conclusions

This study applied a deductive data analytic approach based on the Transactional Theory of Stress and Coping to investigate the stress and coping strategies of Hong Kong university students during the pandemic. The findings revealed four major challenges faced by Hong Kong university students: accumulated negative emotions, health-related anxiety and frequent changes in pandemic-related policies, conflict with family members and challenges in online learning and academic and career development. To cope with the stress arising from these challenges, the most frequently applied coping strategies were seeking social support, problem-focused coping and emphasizing the positive. In particular, students applied the strategies seeking social support, emphasizing the positive and tension reduction to manage their negative emotions; problem-focused coping and emphasizing the positive to deal with health-related anxiety and stress arising from the frequent changes in pandemic-related policies; seeking social support, tension reduction, distancing/detachment and self-isolation/keeping to themselves to handle conflict with family members; and seeking social support, problem-focused coping, emphasizing the positive and distancing/detachment to cope with challenges in online learning and academic and career development uncertainty. Moreover, the findings revealed that more students with a high level of self-perceived resilience employed emphasizing the positive as a coping strategy, while more students with financial difficulties applied tension-reduction coping strategies.

## Data Availability

The data supporting the findings of this study are available upon request from the corresponding author. The data are not publicly available due to ethical concerns regarding the publication of focus group transcripts in a public repository. Specifically, when the data were collected, participants only gave consent for the findings to be published anonymously for educational and research purposes. Making the data public would violate this consent.

## Supplementary Materials

The following supporting information can be downloaded at https://www.mdpi.com/article/10.3390/ijerph22091359/s1. Figure S1. A literature review map on the knowledge gap and key literature covered in this study. Table S1. The three categories, related questions asked before the focus groups, language used in the focus groups, participant codes and the number of participants in each category. Table S2. A summary of the use of coping strategies by research participants and consolidation of research findings. Table S3. Additional illustrative quotes.

## Abbreviations

The following abbreviations are used in this manuscript:

COVID-19	Coronavirus Disease 2019
WCCL	Ways of Coping Checklist
SCSQ	Simplified Coping Style Questionnaire
MCI	Multidimensional Coping Inventory
BACQ	Brief Approach/Avoidance Coping Questionnaire
RSQ	Responses to Stress Questionnaire
CPYDS	Chinese Positive Youth Development Scale

## Appendix A

### Appendix A.1. Reflexivity Statement

The first author is a researcher on positive youth development. Her previous research generally focused on qualitative studies. Prior to this study, she conducted more than 50 individual interviews and focus groups with secondary school teachers and university students on topics including mental health, leadership and teaching and learning effectiveness. These experiences enabled her to be well prepared to engage research participants in meaningful conversation and analyze qualitative data rigorously. However, the potential bias of the first author is that she was an outsider of the group being studied (i.e., university students), so research participants may not be fully open with their feelings and experiences. This bias was minimized by building a rapport with the research participants before and during the focus groups.

The second author is a psychologist with rich experience in conducting both qualitative and quantitative research on positive youth development, family process, scale development, quality of life, program evaluation, addiction and spirituality. He developed the Chinese Positive Youth Development Scale (CPYDS) in 2006 and has contributed to research on youth development for many years. His expertise and experience greatly enhanced the research design, the conceptualization of the theoretical framework and the interpretation of the research findings. The second author served as a “critical friend” to the first author during the data analysis process by challenging the development of codes and themes, enabling the comprehensive analysis of the qualitative data.

### Appendix A.2. Semi-Structured Focus Group Guide

- Challenges faced by students
  1. Can you tell me about the challenges you encountered during the COVID-19 pandemic? Please share your experience.
    - Academic Domain
      - Unfulfilled personal and professional goals due to suspension of learning activities
      - Online learning
      - Disruption of outbound activities
      - Uncertainty in career and employment
      - University life and sense of belonging
      - Technology literacy related to online learning
      - Uncertainty on academic development
    - Personal (including physical, psychological, social and spiritual) Domain
      - Physical health (e.g., illness, lack of exercise, dry eyes, sleep problems, body pain)
      - Psychological (e.g., negative emotions, feeling nervous/anxious/worries/depress/hopelessness, fear of going out, bored, fatigue)
      - Social (e.g., loneliness, lack of peer support, friendship, peer relationship, organization)
      - Spiritual (e.g., reflection on/ rethinking life, human relationship, living, etc.)
      - Have you reflected on your life meaning during the pandemic?
    - Family Domain
      - Competition to use family resources (e.g., Wi-Fi, space, furniture)
      - Increased family conflict
      - Financial hardship (e.g., unemployed/underemployed/business closure/rent/loan)
      - Role change (e.g., take care of siblings/elder/sick family member when learning)
      - Family members/relatives/friends suffering from the COVID-19
    - Community Domain
      - Discrimination (e.g., test-positive/under quarantine/travel history)
      - Community sentiment (e.g., measures and policies imposed by the government, performance of officials/members of Legco, experts)
    - Other examples of challenged experienced by the participants
- Coping with Challenges
  1. How did you deal with the challenge(s) and the feeling(s) (e.g., stress, negative feelings)? Why did you choose this method? Was it useful? Please share with us your experience.
- Review
  1. Do you think the pandemic has helped you to grow better? Why or why not?
  2. What lessons have you learned from the pandemic?
