# Supplementary material for: Stress and Coping Strategies of Hong Kong University Students During the COVID-19 Pandemic: A Qualitative Study
Source: Int J Environ Res Public Health. 2025 Aug 29;22(9):1359. doi: 10.3390/ijerph22091359 (PMC12470091; doi:10.3390/ijerph22091359)
Supplement: Supplementary file 1 [file ijerph-22-01359-s001.zip › ijerph-3751392-supplementary.pdf]

Supplementary Figure S1. *A Literature Review Map on the Knowledge Gap and Key Literature Covered in this Study*

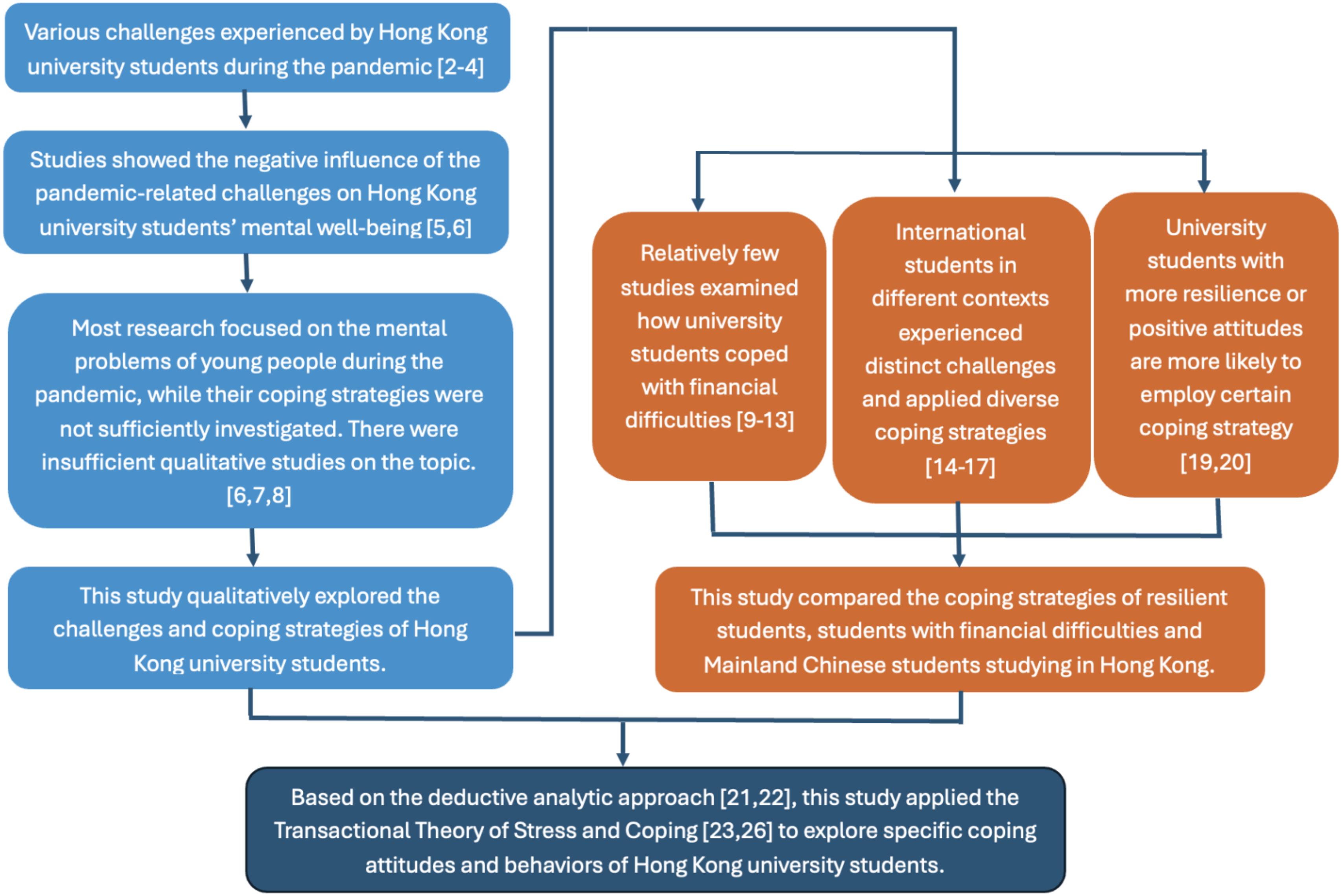

Supplementary Table S1. *The Three Categories, Related Questions Asked before the Focus Groups, Language used in the Focus Groups, Participant Codes and the Number of Participants in Each Category*

Categories	Related Questions Asked Before the Focus Groups	Language Used in the Focus Groups	Participant Codes (Number of Participants)
Students with financial difficulties	Did you or your family face financial difficulties during the pandemic?	Cantonese (Chinese)	F-A-2, F-A-3, F-A-4, F-A-6, F-B-1, F-B-2, F-B-4, F-B-6, F-B-7, F-B-8, F-C-1, F-C-2, F-C-3, F-C-4, F-C-5, F-C-6, F-C-7, F-C-8, F-C-9 (*n* = 19)
Students who coped well	Despite the difficulties I have faced during the pandemic, I consider myself to have coped well. Do you agree with this statement?	Cantonese (Chinese)	C-A-1, C-A-2, C-A-4, C-A-5, C-A-6, C-A-7, C-B-1, C-B-2, C-B-3, C-B-4, C-B-5, C-B-6, C-B-7, C-C-1, C-C-2, C-C-3, C-C-4, C-C-5, C-C-6, C-D-1, C-D-2, C-D-4 (*n* = 22)
Mainland Chinese students studying in Hong Kong	Are you a local, mainland, or international student?	Mandarin (Chinese)	M-A-1, M-A-2, M-A-3, M-A-5, M-A-6, M-A-7, M-A-8, M-B-4, M-B-5, M-B-6, M-B-8, M-B-9, M-C-4, M-C-5, M-C-7 (*n* = 15)
Total number of participants			56

Supplementary Table S2. *A Summary of the Use of Coping Strategies by Research Participants and Consolidation of Research Findings*

The Eight Ways of Coping Scales [23]		
**Items in each scale**	**Participant code**	**Research Findings**
**Problem-focused coping (11 items) (*n* = 24)**		
**1. I go over in my mind what I will say or do.**	**C-C-1 (*n* = 1)**	• Research participants had a high level of anxiety because of various health risk related to the COVID-19. They applied problem-focused coping strategies when facing the uncertainty related to COVID-19 infection and applying effective preventive measures.<br>• Research participants faced various challenges related to online learning. They applied problem-focused coping by finding different ways to resolve related problems.<br>• The pandemic decreased students' opportunities for face-to-face social interaction, meeting new friends and expanding their social circles at university. This negatively impacted their careers and personal development. They tried to resolve this problem by employing problem-focused coping.
**2. Stand my ground and fight for what I want.**	/	
**3. I know what has to be done, so I am doubling my efforts to make things work.**	**C-C-4, C-D-4, F-C-7 (*n* = 3)**	
**4. Come up with a couple of different solutions to the problem.**	**C-A-1, C-A-2, C-A-4, C-A-7, C-C-6, F-B-8, F-C-1, M-A-3 (*n* = 8)**	
**5. I try not to act too hastily or follow my first hunch.**	/	
**6. I'm making a plan of action and following it.**	**C-A-5, M-C-5 (*n* = 2)**	
**7. I try to see things from the other person's point of view.**	/	
**8. I try to keep my feelings from interfering with other things too much.**	**C-C-2, C-C-3, F-B-7, M-C-4 (*n* = 4)**	

**9. Change something so things will turn out all right.**	**C-A-5 (*n* = 1)**	
**10. I try to analyze the problem in order to understand it better.**	**C-B-7, C-C-5, F-C-8, M-B-8, M-B-9 (*n* = 5)**	
**11. Draw on my past experiences; I was in a similar situation before.**	/	
**Emotion-focused coping (*n* = 46)**		
**Wishful thinking (5 items):**		
**1. Wish that I can change what is happening or how I feel.**	/	
**2. Wish that the situation would go away or somehow be over with.**	/	
**3. I daydream or imagine a better time or place than the one I am in.**	/	
**4. Have fantasies or wishes about how things might turn out.**	/	
**5. Hope a miracle will happen.**	/	
**Distancing/Detachment (6 items): (*n* = 6)**		
**1. Try to forget the whole thing.**	**F-B-7 (*n* = 1)**	• Some research participants applied the coping strategy of distancing/ detachment to handle conflict with family members. Although researchers usually regard it as a more passive way of coping, findings from this study showed that it could be effective for young people to reduce the stress caused by family conflict.
**2. Go on as if nothing is happening.**	**C-D-2, F-C-3 (*n* = 2)**	
**3. I'm waiting to see what will happen before doing anything.**	**M-A-2 (*n* = 1)**	
**4. Go along with fate; sometimes I just have bad luck.**	/	

5. **I feel that time will make a difference – the only thing to do is to wait.**	/	• For problems related to career development, when students found that there was no immediate solution, they employed the coping strategy of distancing/ detachment, which could act as a kind of escape for them. They would feel better and gain motivation after a short period of escape from the situation.
6. **Accept it, since nothing can be done.**	**F-C-1, M-A-3 (*n* = 2)**	
**Emphasizing the positive (4 items): (*n* = 22)**		
1. **I'm changing or growing as a person in a good way.**	**C-B-3, M-C-4 (*n* = 2)**	• Research participants stressed the significant influence of negative emotions on their mental health, which stemmed from various challenges they faced during the pandemic, including home confinement, travel restrictions and limited social activities. Research participants release these negative emotions by doing something creative. They adjusted their mindsets and did something interesting to cheer themselves up.<br>• Family members contracting COVID-19 caused significant mental pressure on the research participants. The frequent change in COVID-19-related policies created confusion and uncertainty. They applied emphasizing the positive to cope with related stress.<br>• The Mainland Chinese government implemented policies that were different from those in Hong Kong, which significantly affected students who needed to travel between Hong Kong and Mainland China during the pandemic. To cope with the stress and challenges related to these preventive policies in Mainland China, students also applied the coping strategy of emphasizing the positive.<br>• The pandemic brought a lot of uncertainty to the economy and different industries, which increased
2. **Rediscover what is important in life.**	**C-A-2 (*n* = 1)**	
3. **I am inspired to do something creative.**	**C-A-4, C-B-2, C-B-7, C-C-5, F-B-7, F-C-4, F-C-7, F-C-8, F-C-9, M-C-7 (*n* = 10)**	
4. **Look for the silver lining, so to speak; try to look on the bright side of things.**	**C-B-1, C-B-2, C-B-4, C-B-6, C-B-7, C-C-4, C-D-1, C-D-4, M-B-8 (*n* = 9)**	

		students' stress about their future career development. They coped with related stress by applying the coping strategy of emphasizing the positive, which helped them stay motivated even during difficult times.<br>• The students with high level of self-perceived resilience (i.e., students who coped well) frequently applied the strategy of looking for a silver lining and on the bright side. They tried to look on the positive side when facing difficulties and believed that the future would be better.
**Self-blame (3 items):**		
**1. Criticize or lecture myself.**	/	
**2. Realize I brought the problem on myself.**	/	
**3. Make a promise to myself that things will be different next time.**	/	
**Tension reduction (3 items): (*n* = 17)**		
**1. Got away from it for a while; tried to rest or take a vacation. (Including simply going out and going to the nature)**	**C-A-2, C-A-7, C-C-2, C-D-2, F-B-1 (*n* = 5)**	• Because of prolonged periods staying at home, young people may have experienced a lack of physical exercise, which would deteriorate their physical and mental well-being. Students also worried that poor physical well-being may increase their risk of contracting COVID-19, which could negatively affect their academic performance. Various tension-reduction strategies were employed by research participants to resolve related stress, for example, performing physical exercise and getting away from their problems for a while.<br>• Family conflict could arise from the family's financial difficulties during the pandemic. Family
**2. Try to make myself feel better by eating, drinking, smoking, using drugs or medication, etc.**	**F-B-4 (*n* = 1)**	
**3. I jog or exercise. (Including hiking, meditation and breathing exercise)**	**C-A-7, F-A-2, F-A-4, F-A-6, F-B-4, F-C-2, F-C-4, F-C-5, F-C-6, F-C-8, F-C-9 (*n* = 11)**	

		members could quarrel when discussing financial issues or become impatient or nervous about the family's financial situation, leading to more arguments. The research participants applied a mixture of different coping strategies (tension reduction and seeking social support) to handle stress arising from family conflict.<br>• Most students with personal or family financial difficulties chose to perform physical exercise to release their negative emotions. One of the possible explanations was that students with financial difficulties in Hong Kong usually had smaller living spaces at home, so physical exercise released the feeling of being trapped at home for these students.
**Self-isolation/Keeping to themselves (3 items): (*n* = 3)**		
**1. I try to keep my feelings to myself.**	**C-A-6, M-C-5 (*n* = 2)**	• For Mainland Chinese students who did not return home during the pandemic, they really missed their family members in Mainland China and would often call them or send messages to reduce their homesickness. Students also applied the coping strategy of self-isolation/keeping to themselves to handle the negative emotions resulting from homesickness.
**2. Avoid being with people in general.**	/	
**3. Keep others from knowing how bad things are.**	**M-B-6 (*n* = 1)**	
**Mixed problem/emotion-focused coping (*n* = 29)**		
**Seeking social support (7 items):**		
**1. Talk to someone about how I'm feeling.**	**C-A-6, C-D-2, C-D-4, F-A-6, F-B-2, F-B-4, F-B-6, F-C-2, F-C-5, M-A-2, M-B-4, M-B-5, M-B-9, M-C-7 (*n* = 14)**	• Research participants felt a lot of negative emotions during the pandemic. Many were depressed, anxious, frustrated, feared, worried, stressed, angry,

<table>
<tr><td>2. Accept sympathy and understanding from someone.</td><td>/</td><td rowspan="6">annoyed, irritated and hopeless. Seeking social support, including talking to their family members and friends about their feelings, was an important part of young people's life during the pandemic. It helped to relieve them from the negative feelings that they experienced.
• For students who had mental illness before the pandemic, the pandemic further deteriorated their mental health, and some of them needed to seek professional help.
• Conflict with family members was prominent among the research participants. The findings showed the importance of peer support for reassurance and providing advice to resolve related stress and problems. Sometimes, the situation became so serious that the students needed to seek professional help to deal with family conflict.
• When the Mainland Chinese government lifted the zero-COVID policy, many people were infected with the disease. Mainland Chinese students studying in Hong Kong were worried that their family members and friends in Mainland China would have significant health risk, so they tried to reduce related stress by maintaining close communication.
• Many research participants sought support from friends to deal with online learning challenges. As they made friends with colleagues at the university, they could study together and supervise each other. This kind of peer support helped them to cope with various challenges in online learning.</td></tr>
<tr><td>3. I let my feelings out somehow.</td><td>F-A-3 (n = 1)</td></tr>
<tr><td>4. Talk to someone who can do something concrete about the problem (e.g., psychological counsellor, professional help)</td><td>C-B-5, F-A-2, F-A-4, M-A-8 (n = 4)</td></tr>
<tr><td>5. Talk to someone to find out more about the situation.</td><td>/</td></tr>
<tr><td>6. Ask a relative or friend I respect for advice.</td><td>C-A-6, C-C-3, C-C-4, C-C-6, F-B-2, F-B-6, F-B-8, F-C-1, M-B-6 (n = 9)</td></tr>
<tr><td>7. I pray.</td><td>F-A-2 (n = 1)</td></tr>
</table>

Supplementary Table S3. *Additional Illustrative Quotes*

Themes	Quotes
**Seeking Social Support to Manage Negative Emotions**	*Whenever I encounter any difficulties or challenges, especially during the COVID-19 pandemic, I have been quite lucky to have good friends by my side. I have some good friends. We know each other very well, and we could cheer each other up. We let each other know: although it is difficult now, we are not facing these difficulties alone. We can rely on and support each other. I think this is like a positive coping mechanism: having some friends who can accompany me, willing to share my pain, and willing to listen to me.* (Mainland Chinese student, Group A, M-A-2)
	I would talk to my family members because I usually talk to them about my worries and pressure. (Student with financial difficulties, Group A, F-A-6)
**Emphasizing the Positive to Manage Negative Emotions**	*I have learned how to entertain myself at home. Staying at home does not always make me feel depressed or trapped. I have found lots of ways to cheer myself up.* (Student with financial difficulties, Group C, F-C-4)
	Why did I choose to tidy my home? First, it was for killing time. Second, I like to buy things. I like to buy a lot of things. I was too busy in the past and didn't have time to organize my things. However, during the pandemic, I have more time to tidy my home. After tidying my home, I feel more comfortable and I see the good things in my life. So, this is a way to relieve stress. (Student with financial difficulties, Group C, F-C-9)
**Using Tension Reduction to Manage Negative Emotions**	*I decide to do home workout to release the negative feelings because doing exercise can relieve our unhappy emotions. However, because of the government's preventive measures, many gyms and fitness centers were closed, so many people went hiking. I don't like being in a crowded space, so I choose to stay at home.* (Student with financial difficulties, Group C, F-C-4)
	I really like to do exercise. In short, as long as I can do exercise, then everything is fine. If you require me to wear a mask to jog or hike, I am totally fine with that. As long as you allow me to go out, then it is fine and I don't have a lot of problem. (Student with financial difficulties, Group C, F-C-6)
**Problem-Focused Coping to Deal with Health-Related Anxiety and Stress Arising from the Frequent Change in Pandemic-Related Policies**	*Sometimes our anxiety comes from things that are unclear, for example, the pandemic. We may worry about what to do after getting infected, or what symptoms we will have. If we don't have the information, we may feel very anxious about the pandemic. So, knowing some methods to relieve COVID-19 symptoms could be helpful in reducing stress.* (Mainland Chinese student, Group B, M-B-9)

**Emphasizing the Positive to Deal with Health-Related Anxiety and Stress Arising from the Frequent Change in Pandemic-Related Policies**	*I agree that we need to adjust our mentality to deal with the pandemic. For example, we could pay less attention to the news and try to discover interesting local attractions. Although everyone is trapped in Hong Kong, we must maintain an optimistic and positive attitude. Because this is a global challenge, only through unity can we tide over the difficulties. We need to change our mindset to adapt. We should not pay too much attention to the news, but we should discover the good things about Hong Kong. This is the time for growth. We must face this challenge but still try to move on with life. The key is to change our mindset.* (Student who coped well, Group B, C-B-3)
	The pandemic has made me more resilient and less concerned. I have learned to accept various situations and changes, make better balance of my work and leisure time, and strengthen my ability. (Mainland Chinese student, Group B, M-B-8)
**Seeking Social Support to Handle Conflict with Family Members**	*I talked to my friends about what was going on at home and in school. But it felt like I was escaping reality instead of facing the challenges.* (Student who coped well, Group D, C-D-2)
**Using Tension Reduction to Handle Conflict with Family Members**	*We had conflict in the family, and our relationships have deteriorated. My coping method was to put it aside for the time being and adjust my mentality so that I would not be affected by the problem.* (Student who coped well, Group C, C-C-2)
**Distancing/Detachment to Handle Conflict with Family Members**	*For family and social issues, I just endured it. There was nothing I could do about it, and the problem still existed. [You took all these on your own, didn't you?] Yes.* (Student with financial difficulties, Group C, F-C-1)
**Self-Isolation/Keeping to Themselves to Handle Conflict with Family Members**	*I agree that talking to friends about the problems I encountered will work. However, it might not be a good idea to talk too much to friends about my difficulties because the problems are often continuous and may last for a long time. So, I don't want to bother my friends over and over again.* (Mainland Chinese student, Group B, M-B-6)
**Seeking Social Support to Cope with Challenges in Online Learning, Academic and Career Development**	*When encountering a problem, I usually try to solve it myself or find a solution online. I would also combine my own daily experience to find a solution to the problem. If I can't solve the problem by myself using online resources, I would ask friends around me for their opinions.* (Student who coped well, Group C, C-C-6)
**Problem-Focused Coping to Cope with Challenges in Online Learning, Academic and Career Development**	*I think it is all about flexibility. For example, for learning, we have been using online learning [for a period of time]. If I don't understand something, I would watch the lecture recording a couple times more. If I don't understand certain part, I would watch the lecture recording again or ask my colleagues and professors at the university.* (Student with financial difficulties, Group C, F-C-1)

	When I have questions that I don't understand or don't know how to ask properly, I would keep emailing my professors for answers. As a senior student, I think it is my responsibility to go back to school and ask my professors for answers to complicated questions until they are resolved. (Students who coped well, Group A, C-A-7)
**Emphasizing the Positive to Cope with Challenges in Online Learning, Academic and Career Development**	*The pandemic cannot be completely controlled, so the key is to adjust our mentality and slowly eliminate our fear. Only by paying attention to the pandemic, focusing on self-protection, and changing our mentality and way of thinking, we could better encounter the problems in the pandemic.* (Students who coped well, Group B, C-B-1)
**Distancing/Detachment to Cope with Challenges in Online Learning, Academic and Career Development**	*If there was no way to resolve the problem, I would sleep on it instead of feeling anxious. Because if the problem was not something I could solve, I would rather be easy on myself. If the problem was not something I could change, it would be useless for me to be anxious and to annoy myself.* (Mainland Chinese student, Group A, M-A-3)
